# Advancing from scalar to vectorial liquid crystal holography: a paradigm shift

**DOI:** 10.1038/s41377-024-01538-7

**Published:** 2024-08-23

**Authors:** Yan Li, Shin-Tson Wu

**Affiliations:** 1 Department of Electronic Engineering, Shanghai Jiao Tong University, Shanghai, 200240 China; 2 College of Optics and Photonics, University of Central Florida, Orlando, FL 32816 USA

**Keywords:** Optical materials and structures, Optical manipulation and tweezers

## Abstract

A versatile and tunable vectorial holography is demonstrated based on single-layer single-material liquid crystal superstructures. This novel approach advances the process from scalar to vectorial holography, opening new opportunities for advanced cryptography, super‑resolution imaging, and many other tunable photonic applications.

Holography, with its capability of complete reconstruction of light wavefronts, is promising for diverse applications including displays^1–3^, data storage^4,5^, and optical encryption^6–8^. Traditional scalar holography is mainly focused on the manipulation of light amplitude in the far field, while vectorial holography controls both polarization and amplitude in a spatially varying fashion as illustrated in Fig. 1. Hence, vectorial holography allows for finer light field control, offering richer information capacity and advanced functionalities. The realization of vectorial holography often relies on metasurfaces which could engineer the waveform of electromagnetic waves with an unprecedented level of precision. However, being static, metasurfaces could not provide the flexibility and tunability desired for dynamic photonic applications. Liquid crystal (LC) is a self-organized soft material with both optical and dielectric anisotropies, providing a large-range dynamic control under the application of an electric field^9–12^. Integrating LCs with metasurfaces provides for some degree of tunability^2,13,14^. However, the tunable phase retardation induced by LC typically remains uniform, lacking spatial variation.

**Fig. 1:**
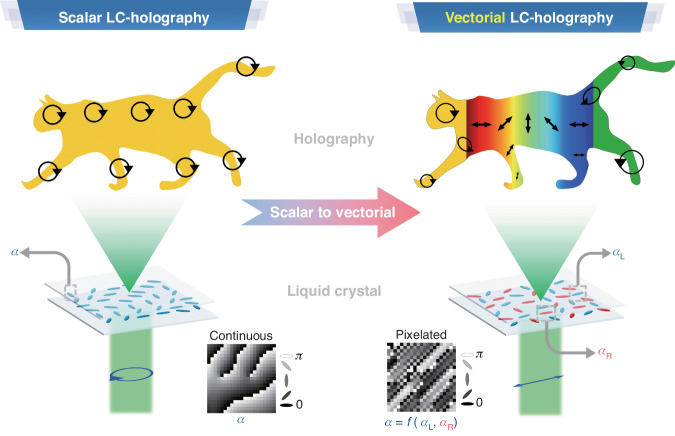
Schematic illustrations of the advancing from scalar to vectorial LC holography

In a recent publication in *eLight*^15^, the team from Nanjing University and National University of Singapore developed a novel encoding method based on helicity-multiplexed pixelated LC superstructures to display versatile and tunable vectorial holography, where both polarization and amplitude can be controlled independently at different positions. This is the first demonstration of vectorial LC holography using a single-layer single-material LC, as opposed to using metamaterials combined with LCs or employing complex optical systems where one of the devices includes LCs.

The LC superstructure is comprised of spatially multiplexed phase holograms encoded for the lefthanded circular polarization (LCP) and the righthanded circular polarization (RCP), respectively, arranged in a checkerboard pattern as Fig. 1 depicts. The phase of the holograms is achieved by designing the LC director’s orientation at different locations based on the geometry phase theory^16–18^. The blue and red ellipsoids shown in the LC holograms represent the LC directors functioning for LCP and RCP, respectively. By optimizing the two holograms, one can modulate the far-field amplitudes (*A*_L_, *A*_R_) and phases (*φ*_L,_ *φ*_R_) of the LCP and RCP components independently. Since *A*_L_, *A*_R_, and the phase difference between LCP and RCP ∆*φ* = *φ*_L_ − *φ*_R_ jointly determine the polarization state, arbitrary polarization state distribution in the far field can be achieved as well.

With the capability of independent and programmable polarization and amplitude control, such LC superstructures enable the realization of vectorial LC holography. The researchers employed a two-loop-iteration modified Gerchberg-Saxton algorithm to generate the helicity-multiplexed LC holograms and demonstrated a vectorial clock (binary engineering of polarization and amplitude) and vectorial lunar phases (continuous engineering of polarization and amplitude), with satisfactory qualities. Also, a vectorial LC‑holographic video was implemented by leveraging the dynamic tunability of LC superstructures.

This work marks the first-ever prototype of lithography-free single-layer single-material LC vectorial holography. With the ability to synthesize full vectorial optical fields, this novel approach greatly enhances the capacity for information encoding, thus spanning multiple cutting-edge fields and offering transformative potential across various applications. It advances the process from scalar to vectorial holography and may display paradigm-shift opportunities for next-generation cryptography, super‑resolution imaging, quantum optical communications, advanced optical data storage, and other related vectorial optical domains.
